# Enhancing Surface Sensing Sensitivity of Metallic Nanostructures using Blue-Shifted Surface Plasmon Mode and Fano Resonance

**DOI:** 10.1038/s41598-018-28122-5

**Published:** 2018-06-27

**Authors:** Kuang-Li Lee, Chia-Chun Chang, Meng-Lin You, Ming-Yang Pan, Pei-Kuen Wei

**Affiliations:** 1 Research Center for Applied Sciences, Academia Sinica, 128, section 2, Academia Road, Nangkang, Taipei 11529 Taiwan; 2 Department of Optoelectronics, National Taiwan Ocean University, Keelung, 20224 Taiwan; 3 Institute of Photonics Technologies, National Tsing Hua University, Hsinchu, 30013 Taiwan; 4 Institute of Biophotonics, National Yang-Ming University, Taipei, 11221 Taiwan

## Abstract

Improving surface sensitivities of nanostructure-based plasmonic sensors is an important issue to be addressed. Among the SPR measurements, the wavelength interrogation is commonly utilized. We proposed using blue-shifted surface plasmon mode and Fano resonance, caused by the coupling of a cavity mode (angle-independent) and the surface plasmon mode (angle-dependent) in a long-periodicity silver nanoslit array, to increase surface (wavelength) sensitivities of metallic nanostructures. It results in an improvement by at least a factor of 4 in the spectral shift as compared to sensors operated under normal incidence. The improved surface sensitivity was attributed to a high refractive index sensitivity and the decrease of plasmonic evanescent field caused by two effects, the Fano coupling and the blue-shifted resonance. These concepts can enhance the sensing capability and be applicable to various metallic nanostructures with periodicities.

## Introduction

Label-free surface plasmon resonance (SPR) sensing enables real-time measurements of biomolecular binding affinity and is beneficial to some applications including food safety, environmental monitoring and medical diagnostics^1–4^. Common SPR sensor utilizes the prism coupling to induce the propagation of surface plasmon waves on metal surface. The SPR can also be excited using metallic nanostructures^5–8^. Compared to the conventional SPR sensors, nanostructured-based SPR sensors have some benefits, including no need of prism, simple measurement, small detection volume, and ease of multiple detections^9–20^. Thought nanostructure-based plasmonic sensors have many advantages, improving their surface sensitivities is still an important issue. Among the SPR measurements, the wavelength interrogation is commonly utilized. When biomolecules attached to the surface of SPR sensors, the wavelength shift (*R*) caused by a biolayer is expressed as

1 $$R=m({n}_{a}-{n}_{s})[1-\exp (-2d/{l}_{d})]$$

where *m* is the bulk (refractive index) sensitivity, i.e. a wavelength shift divided by a refractive index change, *n*_*a*_ the adsorbate biolayer refractive index, *n*_*s*_ the refractive index of the bulk solution, *l*_*d*_ the decay length of evanescent field and *d* the biolayer thickness. When the biolayer thickness and refractive indexes of the bulk solution and biolayer are chosen, the wavelength shift is determined by the decay length and refractive index sensitivity. When surface plasmon waves propagate on a metal surface, *l*_*d*_ can be expressed as the following equation^1^:

2 $${l}_{d}=\text{Im}[\frac{\lambda }{2\pi }{(\frac{{\varepsilon }_{d}+{\varepsilon }_{m}}{{{\varepsilon }_{m}}^{2}})}^{1/2}]$$

where *λ* is the resonance wavelength and *ε*_*d*_ and *ε*_*m*_ are the dielectric constant of adjacent dielectric material and metal, respectively. A shorter decay length can be obtained by choosing a shorter resonance wavelength. According to Equation (1), the sensing capability can be improved by increasing the bulk sensitivity and shorting the decay length. On the other hand, for periodic metallic nanostructures, the Bloch wave surface plasmon polariton (BW-SPP) occurs when the Bragg condition is fulfilled. The resonance condition for one-dimensional nanostructure arrays can be expressed as follows^1^:

3 $${\lambda }_{SPR}(n,\,i,\,\theta )=\frac{p}{i}\{\pm \mathrm{Re}[{(\frac{{\varepsilon }_{m}{n}^{2}}{{\varepsilon }_{m}+{n}^{2}})}^{1/2}]-\,\sin \,\theta \}$$

where *P* is a periodicity of nanostructures, *i* a resonance order, *θ* an incident angle, *n* an environmental refractive index and *ε*_*m*_ a dielectric constant of metal, respectively. For normal incidence, the resonance wavelength is close to *P* × *n*. The refractive index sensitivity is mainly determined by the periodicity of nanostructures, that is *m* = *Δλ/Δn* ~ *P*^17,21,22^. A longer period has a higher refractive index sensitivity. However, a short period is preferred for a short decay length. Therefore, there is a trade-off between the bulk sensitivity and decay length for normally incident light.

**Figure 1:**
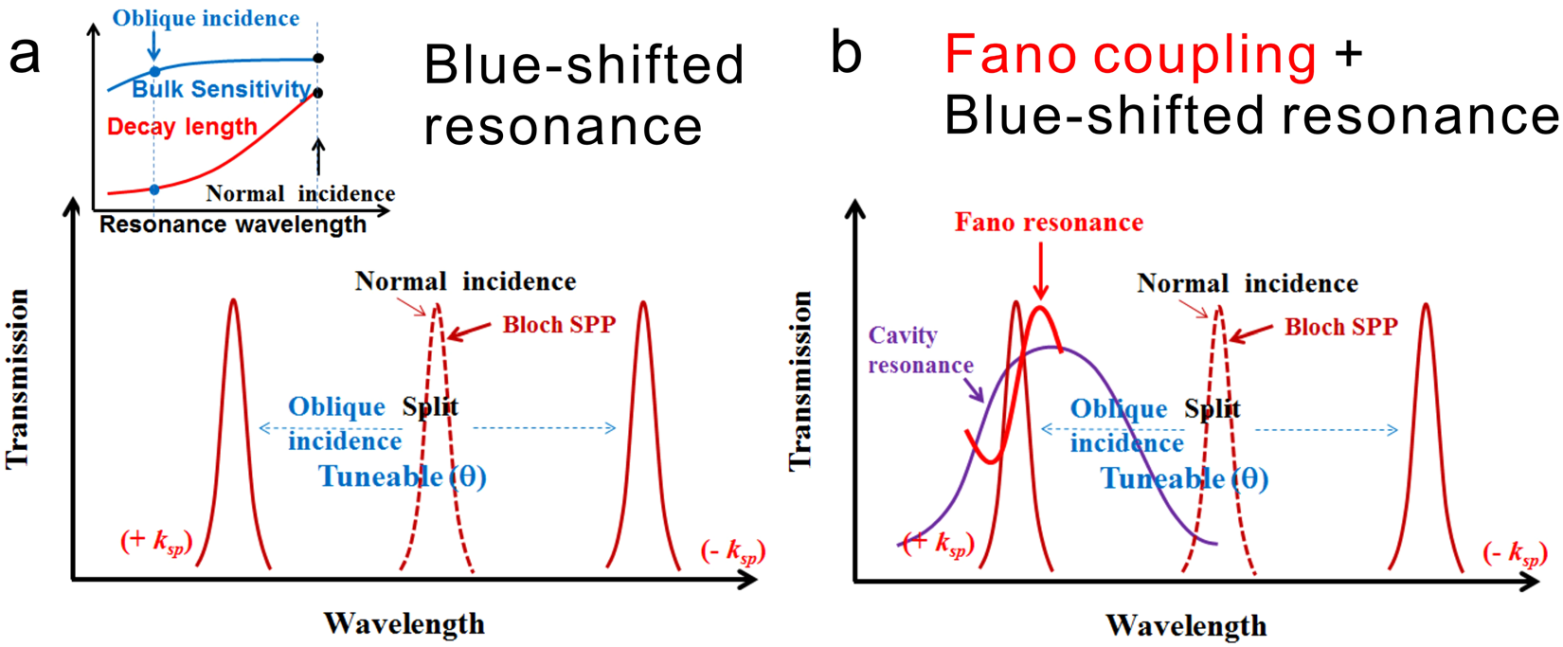
Schematic illustration of the blue-shifted resonance and Fano coupling in periodic metallic nanostructures under oblique-angle incidence. (**a**) Schematic illustration demonstrates the blue-shifted resonance in periodic metallic nanostructures under oblique-angle incidence. The upper panel shows the illustration of bulk refractive index sensitivity and the decay length as a function of the resonance wavelength. (**b**) Schematic illustration demonstrates the blue-shifted Fano resonance in periodic metallic nanostructures under oblique-angle incidence.

According to Equations (2) and (3), the resonance wavelength is a function of the decay length and it is incident angle dependent; the decay length can be shortened through the blue-shifted SPR under oblique-angle incidence^23–26^. On the other hand, the bulk sensitivity is incident angle independent. The surface (wavelength) sensitivity of the periodic metallic nanostructures can be improved by using oblique-angle incidence and increasing the period, which is able to retain the refractive index sensitivity and decrease the decay length simultaneously, as shown in Fig. 1a. Besides, the mode coupling between the long-range surface plasmon polariton (SPP) and localized SPR mode provides an easy way to shorten the decay length. In the metallic nanoslit, the coupling between the SPR and localized cavity mode at a small oblique angle (2°) forms a Fano resonance which can improve the wavelength sensitivity by a factor of 2^27^. In this study, we propose using oblique-angle-induced blue-shifted SPR mode and Fano couplings between the shifted SPR mode and localized cavity mode to improve the wavelength sensitivity of capped silver nanoslits as shown in Fig. 1b. Under oblique angle incidence, the SPR mode is split into backward (−k_SPR_) and forward (+k_SPR_) propagating SPR modes. When the forward-propagating SPR mode (+k_SPR_) is coupled to the broadband cavity mode, an asymmetric Fano resonance^28–30^ is generated. This work combines the Fano coupling effect and blue-shifted resonance in capped silver nanoslits with a longer periodicity, which takes the advantages of large refractive index sensitivity due to long periodicity and short decay length due to short wavelength SPR and Fano coupling effect. The surface sensitivity (or spectral shift) of the coupled mode was enhanced by a factor of 4 when the angle of incidence increased from 0° to 15°. The idea of blue-shifted surface plasmon resonance mode (angle-dependent) in a long-periodicity nanostructure coupled with localized SPR mode (angle-independent) can enhance the wavelength sensitivity and be applicable to various metallic nanostructures with periodicities.

## Results

### Optical properties of 900-nm-period Ti/Ag capped nanoslits with normal and oblique-angle incidence

**Figure 2:**
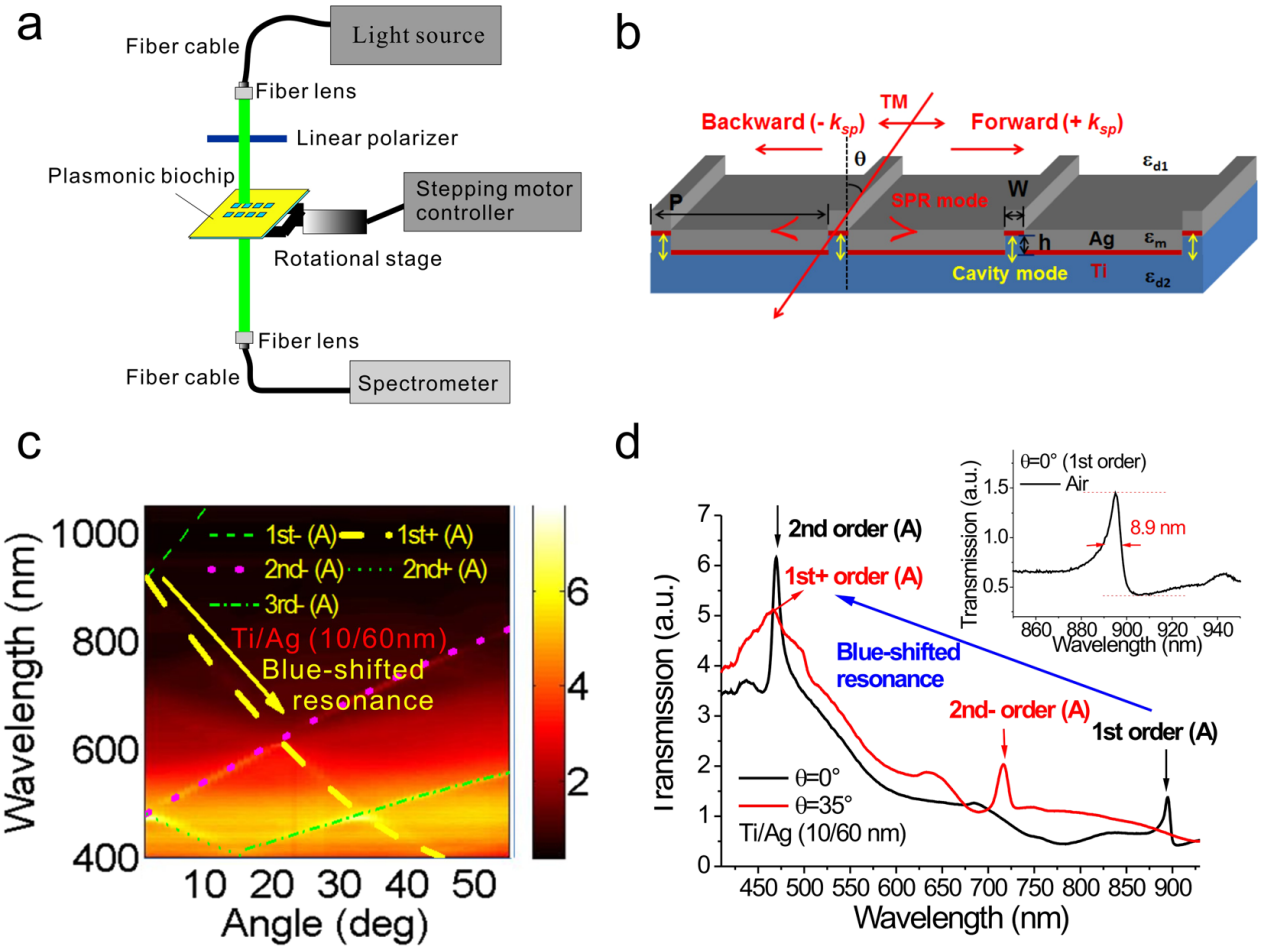
Optical setup and optical properties of 900-nm-period Ti/Ag capped nanoslits with normal and oblique-angle incidence. (**a**) Optical setup for measuring angular transmission spectra. (**b**) Schematic configuration depicts the geometrical parameters of capped nanoslits with a 10-nm-thick titanium and 60-nm-thick silver film and the direction of the TM-polarized incident light. (**c**) Measured angular transmission diagram in air for 900-nm-period capped nanoslit arrays with a Ti/Ag film. The color dashed lines show the theoretical resonance wavelengths for the SPR mode. (**d**) Measured transmission spectra in air at 0° and 35° for 900-nm-period capped nanoslit arrays with a Ti/Ag film.

To verify the concept that wavelength (surface) sensitivity of the periodic metallic nanostructures can be improved using the blue-shifted resonance (see Fig. 1a), we made capped nanoslit arrays with a Ti/Ag film. Figure 2a shows an optical system for measuring transmission angular spectra. Figure 2b shows a schematic configuration that depicts the structure parameters of capped nanoslits with a 10-nm-thick titanium and 60-nm-thick silver film and the direction of the transverse magnetic (TM)-polarized incident light. Figure 2c shows an angular transmission diagram of 900-nm-period capped nanoslit arrays with a Ti/Ag film in air. When the Bragg condition (Equation (3)) is fulfilled, the BW-SPP occurs on the metal surface. It generates a narrowband transmission within the spectrum. As titanium film has a large absorption of surface plasmon wave, the substrate SPR mode is eliminated. It also reduced the resonance condition of cavity mode. Therefore, no Fano coupling occurred in the Ti/Ag structure. The theoretical resonance wavelengths of the SPR modes (the metal/air interface, denoted as “A”) using Equation (3) were shown in Fig. 2c (the color dashed lines). Under oblique angle incidence, the BW-SPP is split into two modes: the forward (+k_SPR_) and backward (−k_SPR_) propagating modes. The results show that the calculated values were close to the measured results. Figure 2d shows the experimental transmission spectra in air at 0° and 35°. The resonance wavelengths were at 894 and 469 nm for 1st and 2nd order SPR modes, respectively. The bandwidth of the 1st order SPR mode for normal-angle incidence was 9 nm as shown in Fig. 2d inset. When the incident angle changed from 0° to 35°, the 1^st^ order resonance mode was split and blue-shifted to 465 nm and the 2^nd^ order mode was red-shifted to 716 nm.

### Surface sensitivity tests of 900-nm-period Ti/Ag capped nanoslits for different incident angles with wavelength interrogation by measuring bovine serum albumin (BSA)/anti-BSA interactions

**Figure 3:**
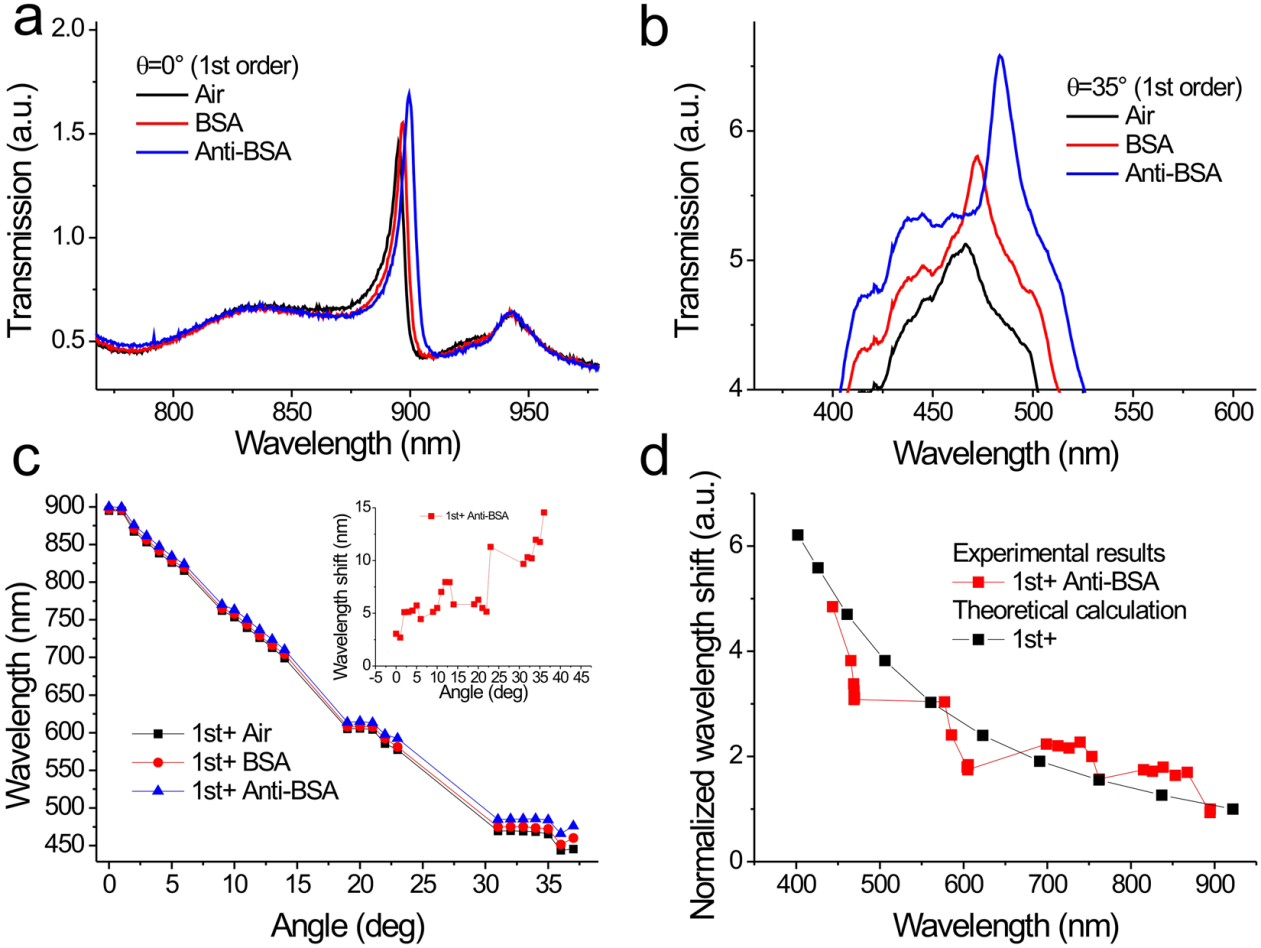
Surface sensitivity tests of 900-nm-period Ti/Ag capped nanoslits for different incident angles with wavelength interrogation by measuring BSA/anti-BSA interactions. Measured transmission spectra of the 1st order resonance mode of the capped nanoslits with a 10-nm-thick titanium and 60-nm-thick silver film at incidence angles of (**a**) 0° and (**b**) 35° in air, BSA (1 mg/mL) and anti-BSA (25 μg/mL). (**c**) Resonance wavelengths as a function of the incident angle in different surface conditions. The insets show the spectral shifts as a function of the incident angle. (**d**) Normalized spectral shift as a function of the resonance wavelength.

We further measured the spectral shifts of the capped nanoslits with a Ti/Ag film caused by the BSA/anti-BSA interactions under normal and oblique-angle incidence. Figure 3a shows the measured transmission spectra of the 1st order resonance at an incident angle of 0° in air, BSA (1 mg/mL) and anti-BSA (25 μg/mL). The BSA (66 kDa) and anti-BSA (150 kDa) caused spectral shifts of 1.9 and 3.0 nm, respectively. When the angle of incidence increased to 35°, the resonance wavelength of the 1st order resonance mode was shifted to 465 nm as shown in Fig. 3b. The wavelength shifts were 7.8 and 11.4 nm, respectively. As the interband transition for a silver film is around 420 nm, the resonance is superimposed with the transmission spectrum of the silver film. To clearly show the resonance, we enlarged the transmission scale. Figure 3c shows the plot of resonance wavelength versus incident angle in different surface conditions. Figure 3c inset shows the plot of spectral shift versus incident angle for anti-BSA binding. Figure 3d shows the comparison of experimental and theoretical normalized spectral shifts as a function of the resonance wavelength for the 1st order resonance mode. The spectral shift of the 1st order resonance with normal angle incidence was chosen as a reference. The experimental spectral shift gradually increased as the resonance wavelength was blue-shifted. It was improved by a factor of 4.8 when the wavelength moved from 894 to 443 nm. Obviously, when the resonance wavelength is shifted to a shorter position, the corresponding decay length will decrease. It causes a larger spectral shift because of the increased overlap between the analyte and the SPR field. To compare with the experimental results, the theoretical spectral shifts were calculated using Equation (3) as showed in Fig. 3d. The trends of the increased spectral shift for the experimental and calculated results were well matched. Therefore, we verified that the SPR mode dominated the surface sensitivity in capped nanoslits with a Ti/Ag film and the blue-shifted resonance was able to improve the surface sensitivity by a factor of 4.8 when the wavelength moved from 894 to 443 nm.

### Optical properties and surface sensitivity tests of 900-nm-period capped silver nanoslits under normal and oblique-angle incidence

**Figure 4:**
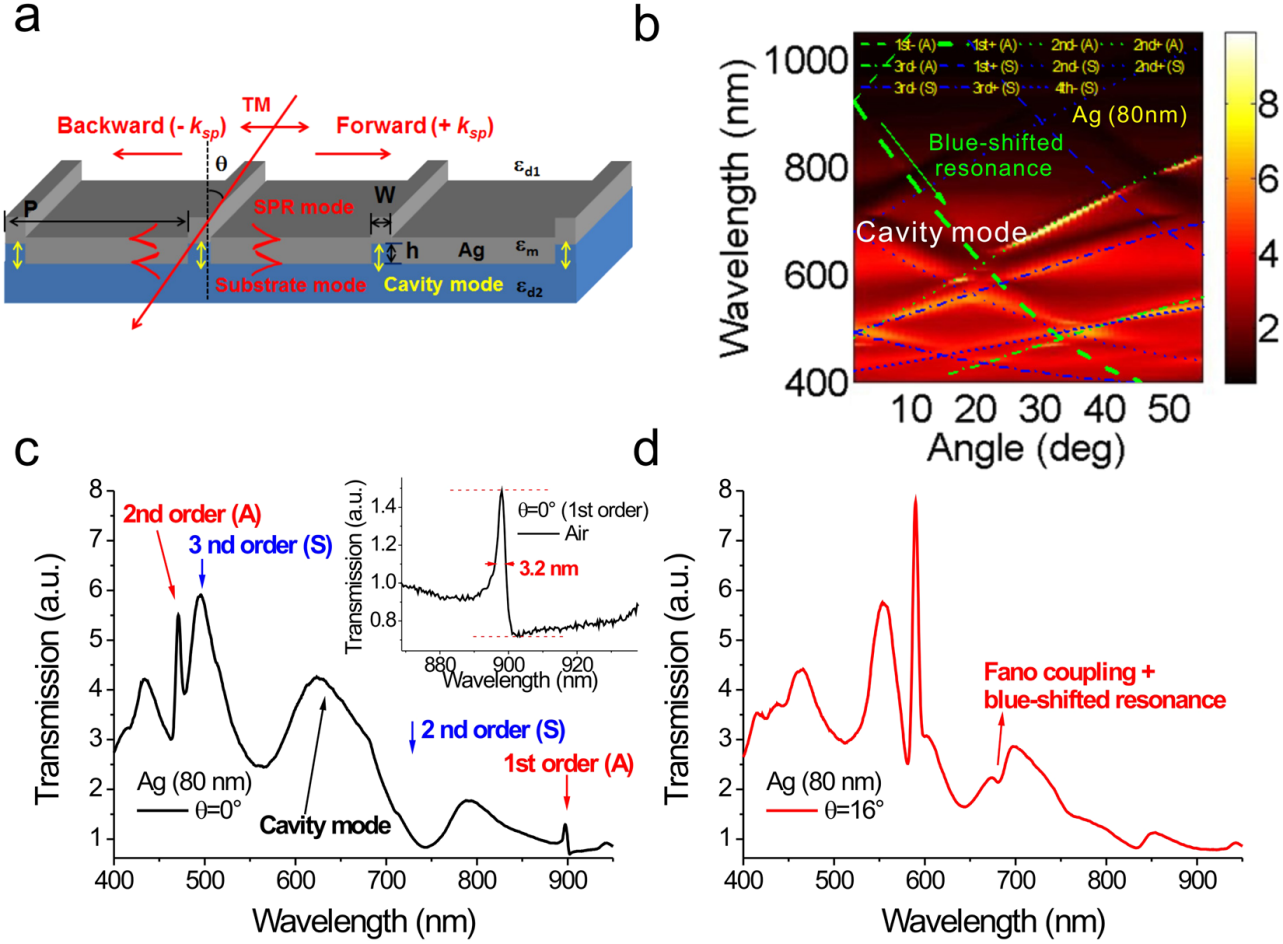
Optical properties of 900-nm-period silver capped nanoslits under normal and oblique-angle incidence. (**a**) Schematic configuration depicting the geometrical parameters of capped silver nanoslits and the direction of the TM-polarized incident light. (**b**) Measured angular transmission diagram of 900-nm-period capped silver nanoslit arrays in air. The green and blue dashed lines show the calculated resonance wavelengths for the SPR and substrate modes, respectively. The measured spectrum of 900-nm-period capped silver nanoslits in air for incident angles of (**c**) 0° and (**d**) 16°. The inset in panel (c) shows the bandwidth of the 1st order mode was 3.2 nm.

To verify the surface sensitivity can be further improved by combing the oblique-angle-induced blue-shifted resonance and the Fano coupling effect. We fabricated capped sliver nanoslit arrays and tested their surface sensitivities. A schematic configuration depicting the geometrical parameters of capped silver nanoslits and the direction of the TM-polarized incident light was shown in Fig. 4a. There are two kinds of resonance modes in the capped nanoslit arrays: the cavity mode in the slit gaps and BW-SPP on the periodic silver surface. The resonance condition for the cavity mode is described by a Fabry-Perot cavity^31^,

4 $$2{n}_{eff}{k}_{0}h+{\varphi }_{1}+{\varphi }_{2}=2m\pi $$

where *k*_0_ is the free space wavelength vector (2π/*λ*_0_), *n*_*eff*_ is the equivalent refractive index in the nanoslit, *ϕ*_1_ and *ϕ*_2_ are the phase shifts at the top and bottom interfaces and *h* is the height of the periodic ridge. Since the reflectance at both interferences is quite small, the cavity mode results in a broadband resonance in the transmission spectrum. The resonance spectrum is independent of the angle of the incident light. On the other hand, the BW-SPP occurs when the Bragg condition (Equation (3)) is fulfilled. It results in a narrowband transmission within the spectrum. Figure 4b shows the measured and theoretical angular transmission diagram of capped nanoslit arrays with a period of 900 nm in air. The green and blue dashed lines in Fig. 4b show the theoretical resonance wavelengths using Equation (3) for the SPR modes (the metal/air interface, denoted as “A”) and substrate (the metal/substrate interface, denoted as “S”) modes, respectively. The calculated values were close to the measured results. Figure 4c shows the measured transmission spectra in air at 0°. The resonance wavelengths were around 625, 897, 471 and 495 nm for the cavity, 1st and 2nd order SPR and substrate modes, respectively. The capped nanoslit array has a metallic cap on the top of nanoslits. It can enhance both the cavity mode and SPR mode and reduce the bandwidth. The bandwidth of the 1^st^ order resonance mode was 3.2 nm as shown in Fig. 4c inset. As the incident angle increased, the 1st order resonance peak was split into two resonance peaks. The split (forward-propagating) SPR mode will couple to the cavity mode when the incident angle is above 13°, which results in a blue-shifted asymmetric Fano resonance as shown in Fig. 4d.

**Figure 5:**
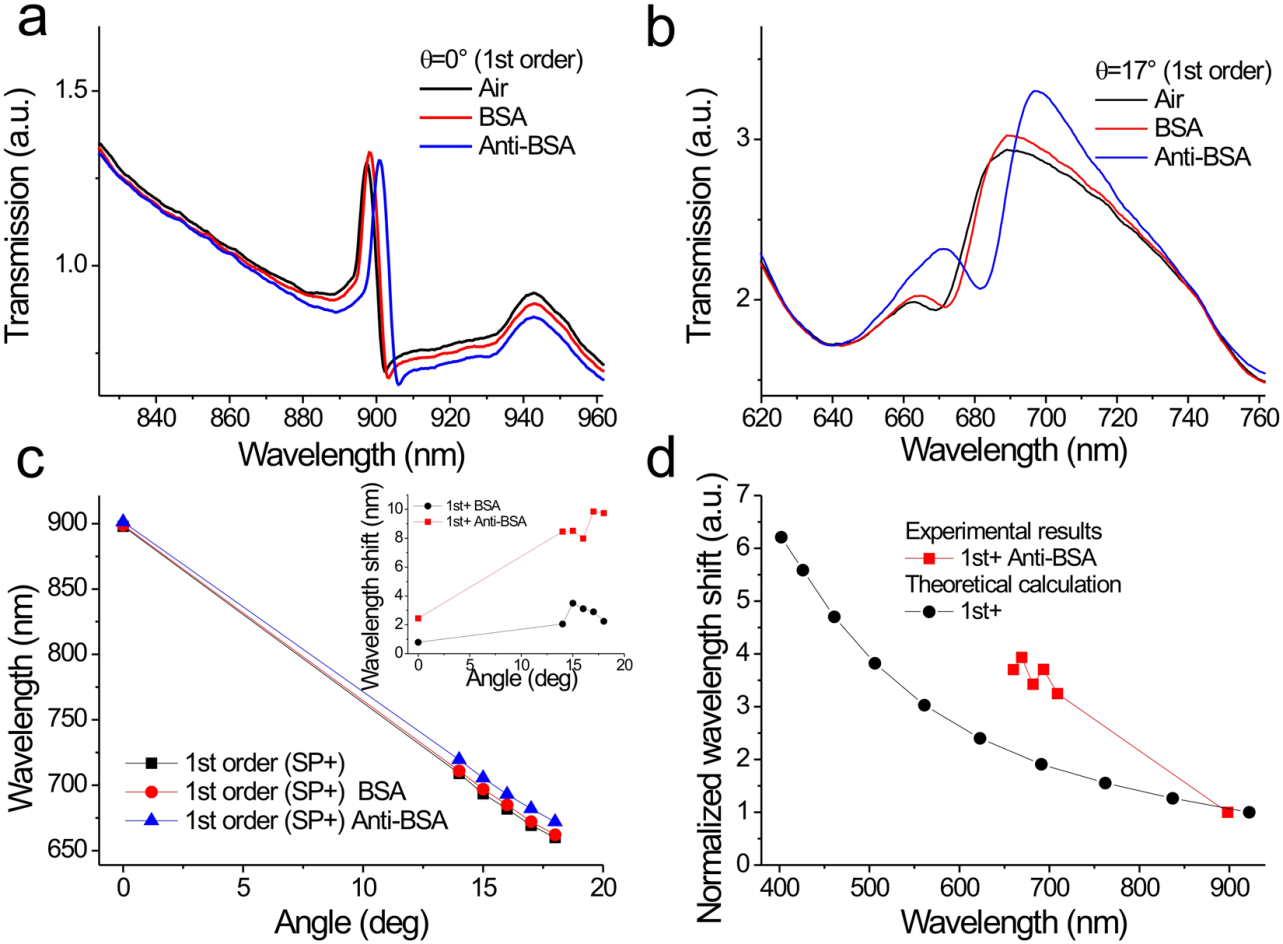
Surface sensitivity tests of 900-nm-period silver capped nanoslits for different incident angles with wavelength interrogation by measuring BSA/anti-BSA interactions. Measured transmission spectra of the 1st order resonance mode of 900-nm-period capped silver nanoslits at incidence angles of (**a**) 0° and (**b**) 17° in air, BSA (1 mg/mL) and anti-BSA (25 μg/mL). (**c**) The resonance wavelengths of the 1st order modes as a function of the incident angle in different surface conditions. The inset shows the spectral shift as a function of the incident angle for the 1^st^ order resonance mode. (**d**) The normalized spectral shift as a function of the resonance wavelength for the 1^st^ order resonance mode.

We further measured the spectral shifts of the capped silver nanoslits caused by the BSA/anti-BSA interactions under different incident angles from 0° to 18°. Figure 5a shows the transmission spectra of the 1st order resonance at an incident angle of 0° in air, BSA (1 mg/mL) and anti-BSA (25 μg/mL). The BSA and anti-BSA caused spectral shifts of 0.8 and 2.5 nm, respectively. When the incident angle increased to 17°, the forward-propagating 1st order resonance mode was coupled to the broadband cavity mode, which generated a blue-shifted asymmetric Fano resonance at a wavelength of 669 nm as shown in Fig. 5b. The spectral shifts increased to 2.9 and 9.9 nm, respectively. Figure 5c shows the plot of resonance wavelength against incident angle for different surface conditions. Figure 5c inset shows the plot of spectral shift against incident angle for BSA and anti-BSA conditions. Figure 5d show the normalized spectral shift as a function of the resonance wavelength. The spectral shifts of the 1st order resonance with normal angle incidence were chosen as a reference. The results show that the spectral shift of the 1st order mode was dramatically increased when the resonance wavelength was blue-shifted to 685 nm. It enhanced by a factor of 4 when the angle changed from 0° to 15°. The significant difference between the experimental and theoretical spectral shifts was observed as shown in Fig. 5d. We attributed the enhanced surface sensitivity to the reduced electromagnetic field decay length caused by the Fano coupling effect. Compare to Ti/Ag nanostructures at the same 685 nm wavelength, the blue-shifted SPR mode enhanced the surface sensitivity by about two times. The additional two times enhancement was attributed to the Fano coupling effect.

### Refractive index sensitivities of capped silver nanoslits with a period of 900 nm for different incident angles

**Figure 6:**
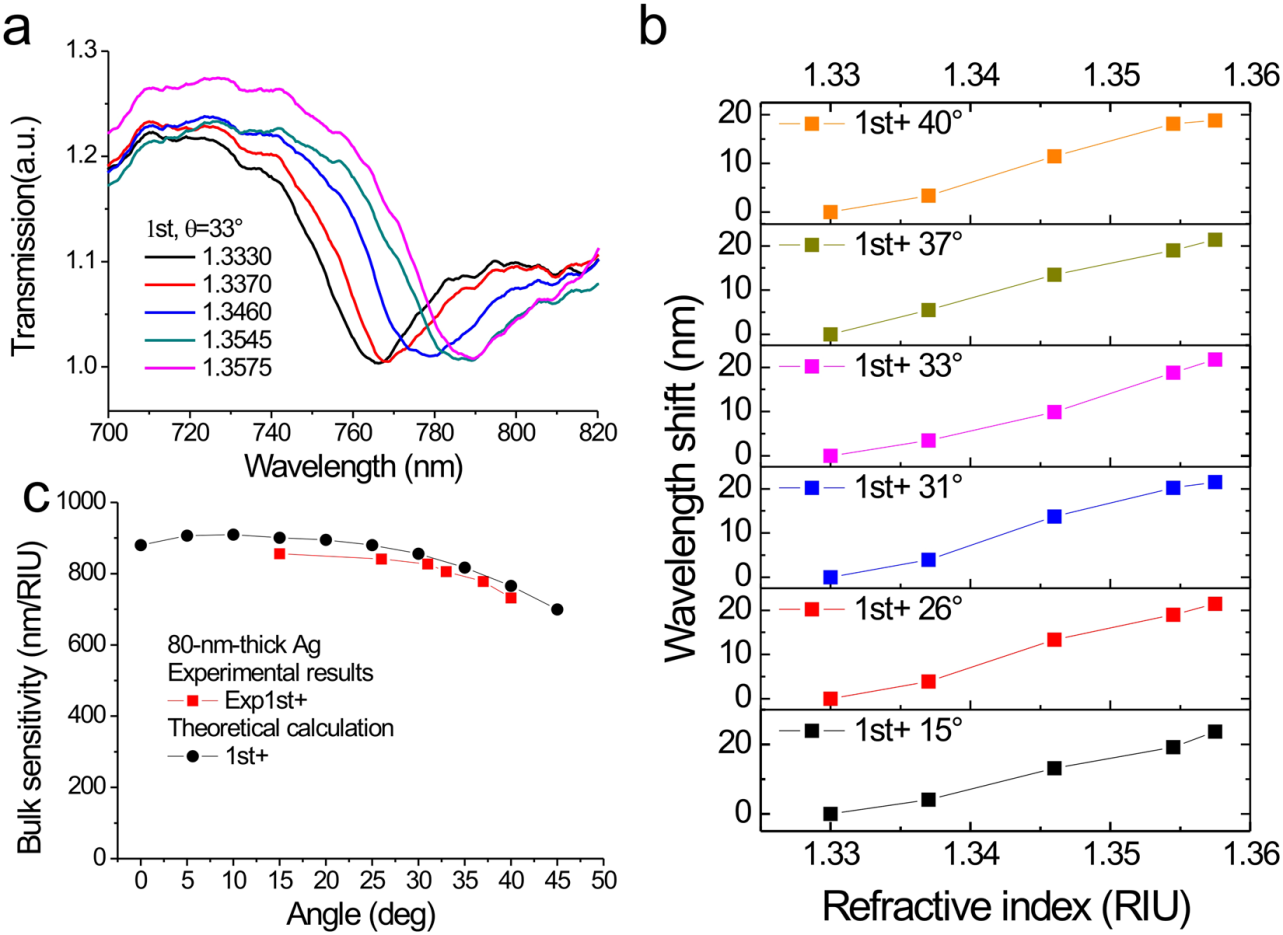
Refractive index sensitivities of capped silver nanoslits with a period of 900 nm for different incident angles. (**a**) Measured transmission spectra in different water-glycerin solutions at an incident angle of 33°. (**b**) The wavelength shift versus the refractive index for the 1^st^ order resonance mode with different incidence angles from 15° to 40°. (**c**) The experimental and calculated bulk sensitivity as a function of the incident angle from 0° to 45° for the 1st order resonance mode.

The SPR surface sensitivity is related to the refractive index sensitivity (or bulk sensitivity) and decay length. To verify the change of refractive index sensitivity, we measured the angular transmission diagram of 80-nm-thick capped silver nanoslits in different water-glycerin solutions. The refractive indexes of the solutions ranged from 1.3330 to 1.3575. Figure 6a shows the measured transmission spectra in different water-glycerin solutions at 33°. The resonances of SPR modes were redshifted with the increase of the refractive index of the solution. Figure 6b shows the wavelength shift versus the refractive index for the 1^st^ order mode at incident angles from 15° to 40°. The peak wavelengths in water were chosen as references. A linear correlation between the wavelength shift and the refractive index of the solution was observed. The results show that for the 1^st^ order resonance mode at 15°, the bulk sensitivity was 856 nm/RIU. It gradually decreased to 732 nm/RIU when the incident angle changed from 15° to 40° as shown in Fig. 6c. The reduced bulk sensitivity was about 14.5%. To compare with the experimental results, the theoretical bulk sensitivities were calculated using Equation (3) as shown in Fig. 6c. The wavelength dependence permittivity of silver is obtained from Palik^32^. For the 1^st^ order mode at an incidence angle of 15°, the calculated bulk sensitivity was 900 nm/RIU. It decreased to 765 nm/RIU when the incident angle increased from 15° to 40°. It results in a decrease of 15%, which is consistent with the experimental result. Therefore, we verified that the enhanced surface (wavelength) sensitivity in capped silver nanoslits as shown in Fig. 5d was due to the decrease of the decay length, caused by the oblique-angle induced Fano resonance.

### Simulations of transmission spectrum and resonance field distributions of capped metallic nanoslits

**Figure 7:**
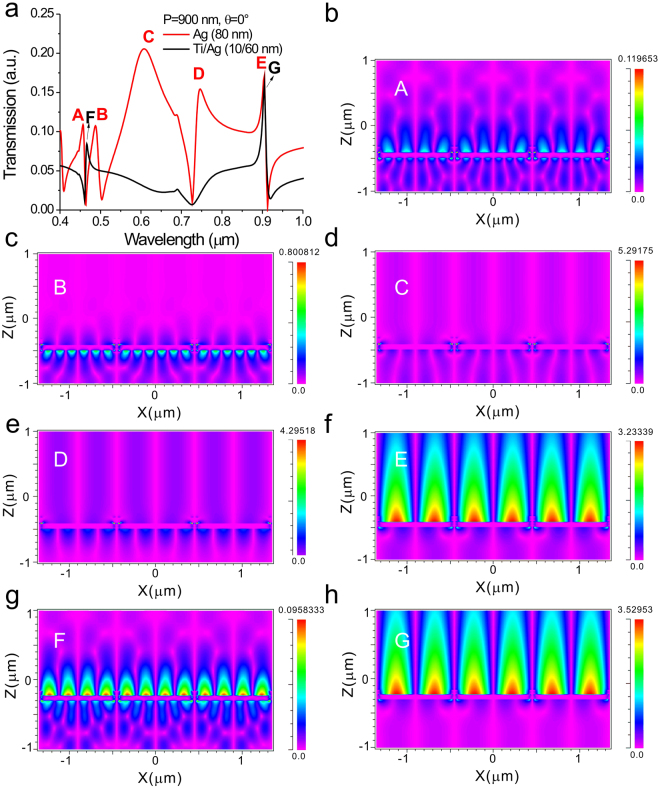
Simulations of transmission spectra and resonance field distributions of capped metallic nanoslits. (**a**) Calculated transmission spectra of the capped nanoslits with a silver film and a Ti/Ag film for normally incident TM-polarized light using FDTD calculations. The simulated absolute E_z_ field distributions for (**b**) peak A, (**c**) peak B, (**d**) peak C, (**e**) peak D, (**f**) peak E, (**g**) peak F and (**h**) peak G.

To further verify the measured results, the calculated spectra of capped nanoslits with a silver film and a Ti/Ag film for normally TM-polarized incident light were shown in Fig. 7a using finite-difference time-domain (FDTD) calculations. The simulated absolute Ez field distributions for the resonance peaks (peaks A–G) were shown in Fig. 7b–h. The wavelengths of peaks A, B, C, D and E are 456, 487, 608, 746 and 904, respectively. They represented the 2^nd^ order SPR mode, 3^rd^ order substrate mode, cavity mode, 2^nd^ order substrate mode and 1^st^ order SPR mode, respectively. Peak F (466 nm) and G (905 nm) in Fig. 7a were the 2nd and 1st SPR modes, respectively. The mode order (i) can be identified from the interference patterns. The number of interference fringe within a period is 2i. For example, the asymmetric Ez field for the 1^st^ order mode (peaks E and G) results in two fringes within a period. For 2^nd^ order modes (peaks A, D, F), there are 4 fringes in a period. For SPR modes, the Ez fields were distributed on the metal/air (peaks A, E, F, G). For the substrate modes, the Ez fields were at metal/substrate interfaces (peaks B, D). For the cavity mode, the electric field was confined in the slits (peak C). The shorter resonance wavelength has a shorter evanescent tail. Compared to the 2^nd^ order SPR mode, the 1^st^ order SPR mode has a long evanescent tail. Obviously, the cavity and substrate modes were depressed for capped nanoslit arrays with a Ti/Ag film. The calculated spectra matched quite well with the experimental data shown in Figs 2d and 4c. Therefore, we attributed the enhanced spectral shift of the capped silver nanoslit array in Fig. 5d to the decrease of the electromagnetic field decay length caused by the short SPR and Fano coupling effect.

**Figure 8:**
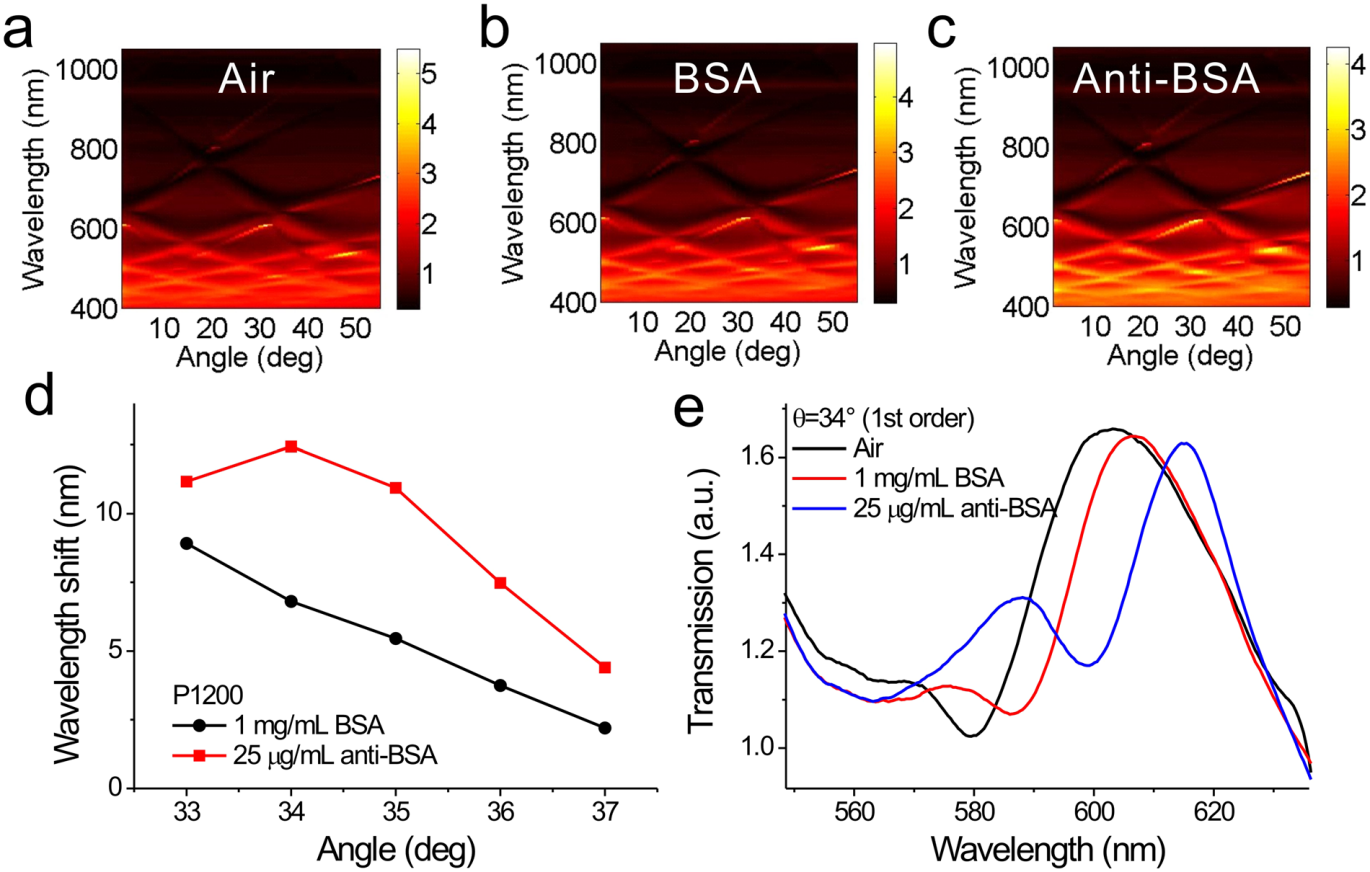
Surface sensitivity tests of 1,200-nm-period silver capped nanoslits for different incident angles with wavelength interrogation by measuring BSA/anti-BSA interactions. Measured angular transmission diagrams of the 1,200-nm-period capped silver nanoslits in (**a**) air, (**b**) BSA (1 mg/mL) and (**c**) anti-BSA (25 μg/mL) for incidence angles from 0° to 55°. (**d**) Spectral shift as a function of the incidence angle. The spectra in air were chosen as references. (**e**) Measured transmission spectra of the 1st order resonance mode in air, BSA (1 mg/mL) and anti-BSA (25 μg/mL) for an incidence angle of 34°.

It was noted that the surface sensitivity can be further increased when the coupled resonance mode forms at a shorter wavelength and nanostructures with a longer period is utilized. Figure 8a–c show the measured angular transmission diagrams of capped silver nanoslits with a period of 1,200 nm in air, BSA (1 mg/mL) and anti-BSA (25 μg/mL), respectively. Figure 8d shows the spectral shift of the 1^st^ order resonance mode as a function of the incidence angle. The spectra in air were chosen as references. Figure 8e shows the measured transmission spectra of the 1st order resonance mode at 34° in different surface conditions. The spectral shifts caused by 66-kDa-sized BSA and 150-kDa-sized anti-BSA were 6.8 and 12.4 nm, respectively. We further estimated the detection limit of the surface mass density of anti-BSA. The surface mass density can be estimated by the thickness, that is, surface mass density (μg/cm^2^) ≈ 0.12 × t (nm)^33^. For 25 μg/mL anti-BSA adsorption, the thickness is 1.9 nm and the surface mass density, 0.22 μg/cm^2^, is estimated^34^. Based on the assumption of linear response and the current spectral resolution (0.3 nm), the detectable surface mass density of the anti-BSA proteins is 5.3 ng/cm^2^ for an incident angle of 34°. Such a sensing capability is better than that of localized SPR, in visible light region, in random nanoholes (15 ng/cm^2^, under a wavelength resolution 0.1 nm)^35^ and SPR, in near-infrared light region, in a two-dimensional nanohole array with a periodicity of 1.53 μm (30 ng/cm^2^, under a wavelength resolution 0.3 nm)^17^. If the spectral resolution (or noise level) is improved to 0.5 × 10^−4^ nm using low-noise high-resolution spectrometer combined with curve fitting and other algorithms^35^ (see Supplementary Information), the detectable surface mass density can be further reduced to 8.8 pg/cm^2^. Such a limit of detection was better than that in the quartz crystal microbalance (QCM) detection technique (2 ng/cm^2^, with a resolution of 0.1 Hz) and in the bulky attenuated total reflection (ATR) sensors using an angular detection method (0.1 ng/cm^2^, with a resolution of 0.1 mdeg)^36^. Besides, such a sensing capability is comparable to that of ultrathin metal-dielectric nanophotonic cavity with phase interrogation (5.1 pg/cm^2^, with a phase noise of 0.5 deg)^37^, but 2-orders of magnitude worse than that of plasmonic metamaterials with topological darkness (0.1 pg/cm^2^ with a phase noise of 0.5 deg)^38^. Compared to intensity, wavelength and angle interrogation, the phase measurement has a higher sensing capability. It was noted that the limit of detection of the proposed approach can be further improved using a high-resolution spectrometer or by increasing the periodicity of the nanostructure and incident angle. However, this method is limited by the propagation length of the surface plasmon wave. As the propagation length for surface plasmon wave (λ = 630 nm) on silver/water interface is 19 μm^39^, the periodicity of the nanostructures should be shorter than the propagation length. In addition, the sensing capability can be further enhanced by depositing a thin capped dielectric layer on the nanostructures, which is able to significantly reduce the decay length by factors of 3–9^40^.

## Discussion

We proposed the use of blue-shifted Fano couplings with oblique-angle incidence to increase the surface sensitivities of periodic metallic nanostructures with longer periodicities (900 nm and 1,200 nm). This method simultaneously maintained the higher bulk sensitivity and greatly reduced the electromagnetic field decay length, resulting in an improvement in the surface sensing capability. In this study, we show an improvement by at least a factor of 4 in surface sensitivities of capped silver nanoslit arrays. It was attributed to the decreased electromagnetic field decay length caused by two effects, the Fano coupling and the blue-shifted resonance wavelength. The split and blue-shifted surface plasmon resonance mode (angle-dependent) in a long-periodicity nanostructure coupled with localized cavity mode (angle-independent) and resulted in a blue-shifted Fano coupling mode. It affected the field distribution and resulted in a reduced decay length for the coupling mode. It is noted that the oblique-angle incidence also enhanced the sensitivity of short-period nanostructures via. Fano coupling effect. However, the short-period nanostructure needs to increase (redshift) the SPP resonance wavelength for the interaction with the cavity mode resonance. It thus limits the enhancement factor. For example, the 500-nm-period capped nanoslit array at an incident angle of 2° had 2.94 and 7.42 nm wavelength shifts for BSA and anti-BSA, respectively^27^. In this work, we used a long-period structure (1,200 nm) and a large oblique angle to blueshift SPP mode to the cavity mode (Fig. 8d). The spectral shifts for BSA and anti-BSA proteins were 6.8 and 12.4 nm, respectively. Both were about two times higher than those in our previous work. The comparisons between short-period and long-period nanostructures are shown in Table 1.

**Table 1:** Comparisons between short-period nanostructure (ref.^27^) and long-period nanostructure (this work) under oblique incidence conditions.

Nanostructure	Long-periodicity capped nanoslits (900 and 1,200 nm)	Short-periodicity capped nanoslits (500 and 520 nm)
Oblique induced shift	SPP mode blueshifted	SPP mode redshifted
Effect	Decreased evanescent length of SPP mode	Increased evanescent length of SPP mode
Sensing enhancement mechanism for small angle	None	Cavity mode coupling (Fano effect)
Sensing enhancement mechanism for large angle	Short SPP evanescent length + cavity mode coupling (Fano effect)	Three mode couplings: substrate mode + cavity mode + SPP mode
Spectral response	Higher wavelength sensitivity, but larger bandwidth	Sharp bandwidth for three mode coupling, high intensity sensitivity
Major enhancement signal	Peak wavelength shift	Resonance intensity change

The figure of merit (FOM), defined as wavelength sensitivity/resonance width, is also an important factor to evaluate the quality of plasmonic sensors. This FOM has a large influence on the intensity sensitivity with intensity interrogation because the narrower bandwidth has a sharper slope, which can improve the intensity sensitivity. However, for wavelength interrogation, the resonance position can be determined with high accuracy even with moderate wavelength resolution (i.e. a broad resonance)^36,37^. It was reported by Lahav *et al*. that a nearly guided wave SPR sensor, a thin silicon top layer on the SPR sensor, can improve the sensitivity by a factor of 4 even if the enhanced shift is associated with enhanced widening of the resonance dip. In our experiment, the similar results were observed for the blue-shifted Fano resonance as shown in Fig. 5. The improved wavelength sensitivity accompanied with a widened resonance. The resonance position can also be accurately determined using Gaussian or parabolic functions.

## Methods

### Fabrication of capped metallic nanoslits

**Figure 9:**
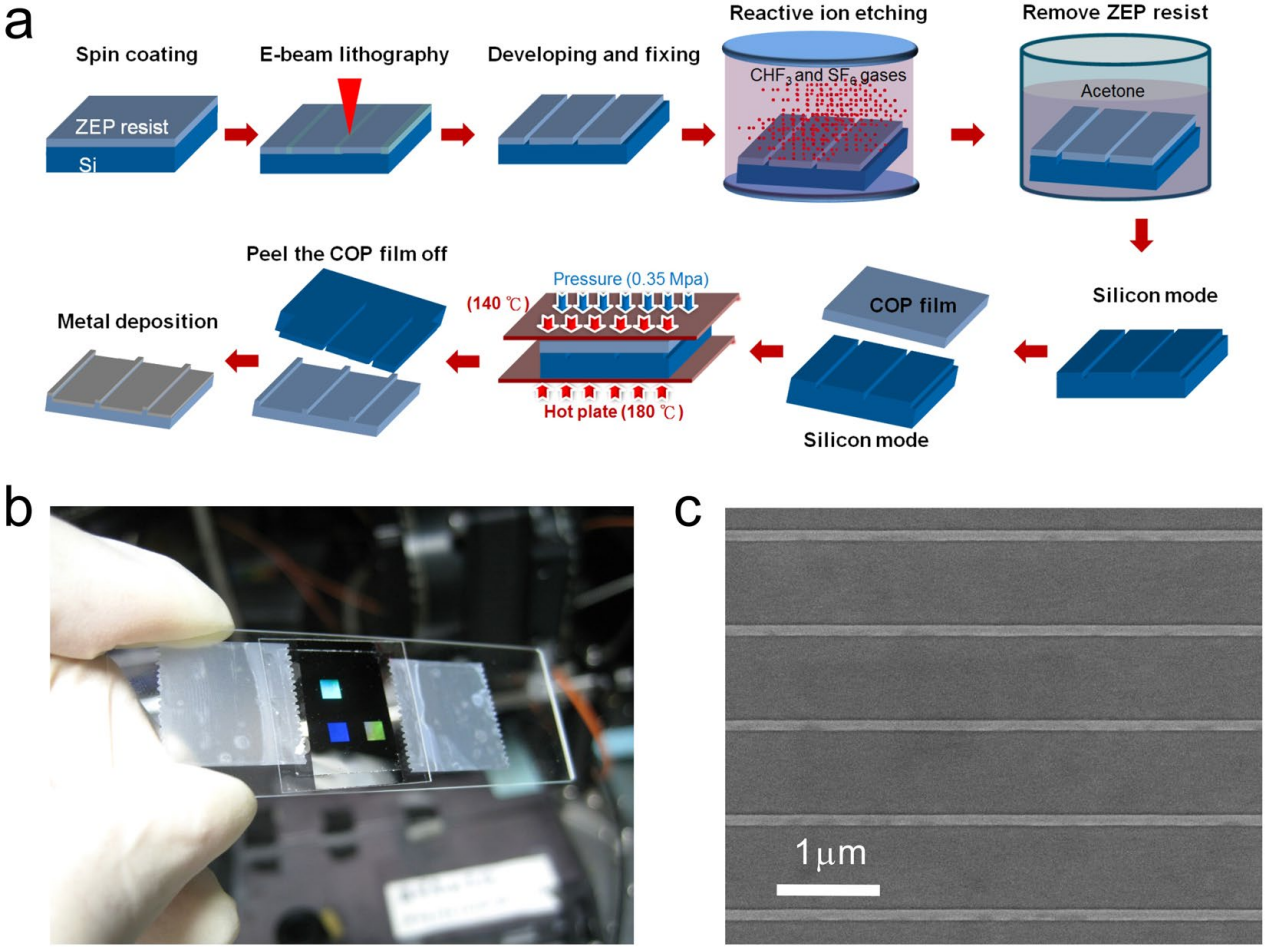
Fabrication of capped metallic nanoslits. (**a**) A flowchart for fabricating capped silver nanoslits. (**b**) Optical and (**c**) SEM images of the capped silver nanoslits. There were 3 nanostructure arrays on the chip and each area was 2 × 2 mm^2^. The ridge width was 80 nm.

Figure 9a shows a flowchart for fabricating capped silver nanoslits. First, a ZEP-520 positive resist with a thickness of 300 nm (Zeon Corp, Japan) was coated on a 4-inch silicon wafer using a spinner. Periodic groove arrays were made onto the resist by electron-beam Lithography (ELS 7000, Elionix, Japan). The width, depth and period of the grooves were 80, 80 and 900 (or 1,200), respectively. The resist structures were transferred to the silicon wafer by dry etching method (Plasmalab 80plus, Oxford Instrument). To remove the residual resist, the wafer was dipped into an acetone solution for a few minutes. The sample was put in deionized water and then blow-dried using a nitrogen flow. The fabricated silicon stamp was then utilized to replicate nanostructures on a 188-µm-thick cyclic olefin polymer (COP) film using hot embossing nanoimprint lithography (EHN-3250, Engineering System Co. Ltd, Japan). The COP substrate and silicon stamp were placed on heating plates. The temperatures of the lower and upper plates were 180 and 140 °C, respectively. A pressing pressure (0.35 Mpa) was applied to the heating plate to press the mold into the softened COP film. The mold and plastic film were then cooled and separated. After depositing a silver film (80 nm) or Ti/Ag film (10 nm/60 nm) on the replicated plastic ridges by an electron beam evaporator (or DC sputter), the metallic nanostructures were made (see Fig. 9b). There were 3 nanostructure arrays on the chip and each area was 2 × 2 mm^2^. Figure 9c shows the scanning electron microscopy (SEM) image of the capped silver nanoslit arrays. The ridge width was 80 nm.

### Angular transmission spectrum measurement

Figure 2a shows an optical system for measuring angular transmission spectra. A laser-driven broadband light source (LDLS™) with fiber-coupled output was connected to a fiber cable. The broadband light was collimated by a fiber lens and its polarization was controlled by a linear polarizer. The polarized light was then incident on the metallic nanostructures. To control the angle of incident light, the nanostructures were put on a rotational stage, controlled by a stepper motor. The transmitted light from the nanostructures was collected by a fiber lens and focused on a fiber cable, connected to a fiber-coupled high performance back-thinned charge-coupled device spectrometer (BWTEK, i-trometer^TM^). The angular transmission spectra of the nanostructures were recorded by simultaneously controlling the stepper motor and spectrometer with a computer. The biological interactions between BSA (Sigma-Aldrich) and anti-BSA (Sigma-Aldrich) proteins were conducted in ultrapure water. BSA proteins were first immobilized on the sensing area by dropping the 1 mg/mL BSA solution (20 μL) for 1 hour. To remove the unbound BSA proteins, the nanostructure was then washed by ultrapure water and blow-dried by a nitrogen flow. After immobilization of BSA proteins, a 25 μg/mL anti-BSA solution (20 μL) was dropped on the chip for 30 min. After protein-protein interactions, the chip was washed by ultrapure water and then blow-dried by a nitrogen flow.

## Electronic supplementary material

Supplementary information
