# Supplementary material for: Enhancing Surface Sensing Sensitivity of Metallic Nanostructures using Blue-Shifted Surface Plasmon Mode and Fano Resonance
Source: Sci Rep. 2018 Jun 27;8:9762. doi: 10.1038/s41598-018-28122-5 (PMC6021451; doi:10.1038/s41598-018-28122-5)
Supplement: Supplementary file 1 — Supplementary information [file 41598_2018_28122_MOESM1_ESM.docx]

**Supplementary Information**

**Enhancing Surface Sensing Sensitivity of Metallic Nanostructures using Blue-Shifted Surface Plasmon Mode and Fano Resonance**

Kuang-Li Lee^1,*^, Chia-Chun Chang^2^, Meng-Lin You^1^, Ming-Yang Pan^1,3^ and Pei-Kuen Wei^1, 2, 4,**^

^1^Research Center for Applied Sciences, Academia Sinica, 128, section 2, Academia Road, Nangkang, Taipei 11529, Taiwan

^2^Department of Optoelectronics, National Taiwan Ocean University, Keelung 20224, Taiwan

^3^Institute of Photonics Technologies, National Tsing Hua University, Hsinchu 30013, Taiwan

^4^Institute of Biophotonics, National Yang-Ming University, Taipei 11221, Taiwan

*[kllee@gate.sinica.edu.tw](mailto:kllee@gate.sinica.edu.tw) and **[pkwei@sinica.edu.tw](mailto:pkwei@sinica.edu.tw)

In our manuscript, we cited a study reported by Dahlin et al^35^ to show that the spectral resolution is possible to be improved to 0.5×10^-4^ nm, which is critically determined by the measurement principle, optical components, and the algorithms employed to analyze the spectral variations. They utilized a thermoelectrically (TE) cooled linear photodiode array, which outperforms the charge coupled device (CCD) by a factor of ~10 in noise levels, and an optical long-pass filter to dynamically measure the dark counts, which can improve the accuracy and long-term stability of the data. To flatten the raw data spectrum and allow values close to saturation to be reached in a wider wavelength region, colored glass filters were introduced. In addition, the centroid (center of mass) of the peak position was tracked with a high-degree polynomial fitting. They demonstrated that the short-term noise levels in centroid is < 5×10^-4^ nm. In our current system with a low-cost miniature CCD-based spectrometer, we utilized 9^th^-order polynomial fitting and calculated the centroid (center of mass) of the dip in the anti-BSA condition (see Fig. S1(a)-(c)). The high-order polynomial fit to the spectral data, *f(x,t)*, is expressed as follows:

$f(x,t)=\sum_{i=0}^{n} P_{i+1}x^{i}$ (1)

where *P* is a vector containing the parameters defining the polynomial and *n* is the degree of the polynomial. The centroid wavelength is calculated from the following equation:

$\lambda_{centroid}(t)=\frac{\int_{\lambda_{s}}^{\lambda_{s}+S} x(f(x,t)-f_{0})dx}{\int_{\lambda_{s}}^{\lambda_{s}+S} (f(x,t)-f_{0})dx}$ (2)

where *f_0_* is a baseline value, *S* is wavelength span, *λ_s_* is the left point of the interval of length *S*. The baseline value is initially chosen as the half-maximum of the dip. *S* and *λ_s_* are determined from *f_0_*= *f(λ_s_)*=*f(λ_s_+S)*. The result shows that the spectral resolution can be reduced from 0.3 nm to 0.07 nm (see Fig. S1(d)). This spectral resolution can be further improved if a high-resolution spectrometer with low-noise, TE-cooled linear detector array and high-stability light source are employed.


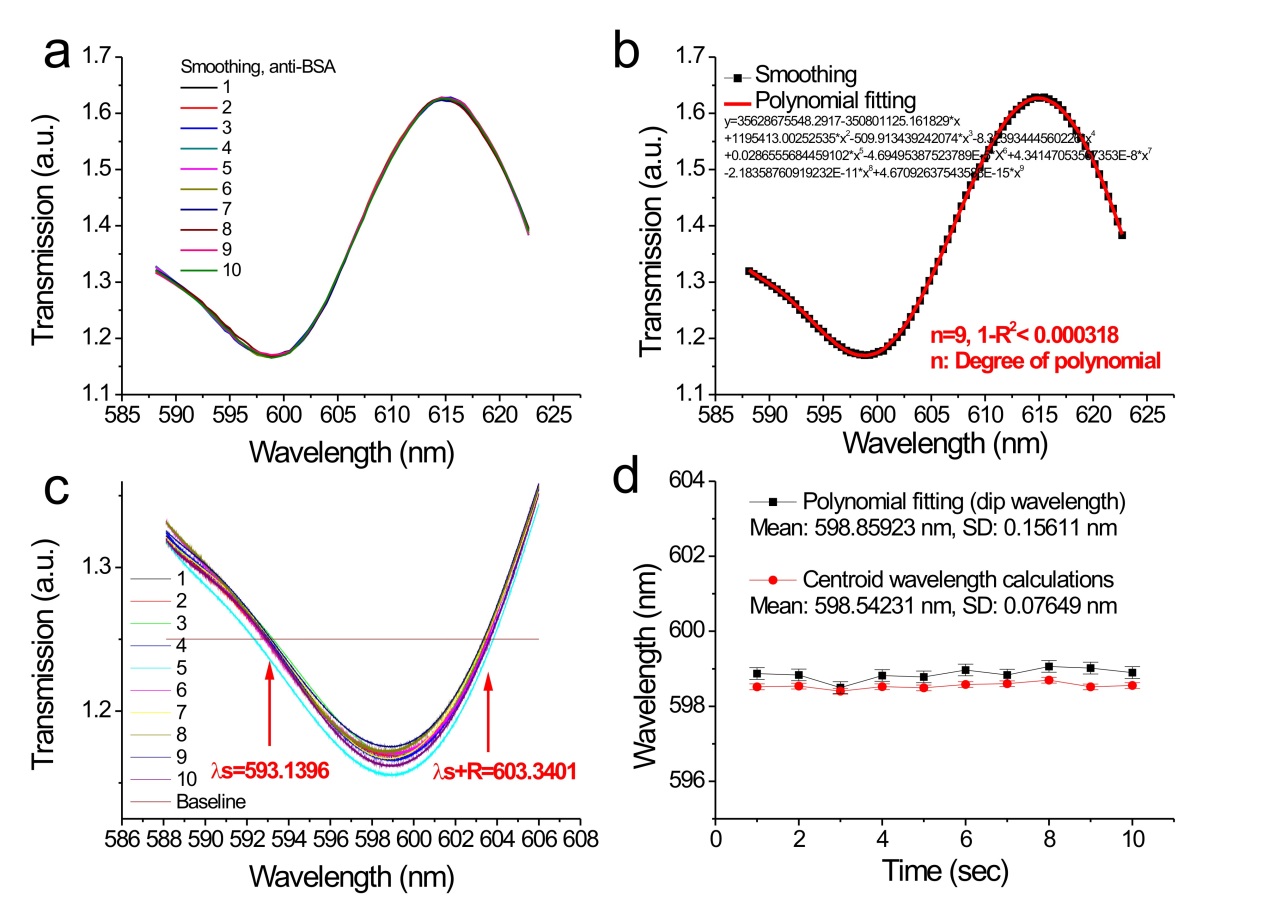


Figure S1. Wavelength resolutions calculated by polynomial fitting and centroid wavelength methods. (a) Enlarged transmission spectra in 25 μg/ml anti-BSA for the time-lapse measurement. (b) 9^th^-order polynomial fitting of the measured spectrum. (c) Enlarged dip spectra for the time-lapse measurement in 25 μg/ml anti-BSA. (d) Calculated dip wavelength as a function of time by polynomial fitting and centroid wavelength methods. The noise levels of the dip wavelength were 0.15 and 0.07 nm for the polynomial fitting and centroid wavelength calculations, respectively.

1. Dahlin, A. B., Tegenfeldt, J. O. & Hook, F. Improving the instrumental resolution of sensors based on localized surface plasmon resonance. *Anal. Chem.* **78**, 4416-4423 (2006).
